# Characteristics and outcomes after out-of-hospital cardiac arrests in individuals with pre-existing psychiatric conditions, compared to those without

**DOI:** 10.1016/j.resplu.2026.101356

**Published:** 2026-05-06

**Authors:** Linnea Gustafsson, Anna Myredal, Clara Hjalmarsson, Lina Holmqvist, Alfred Hjalmarsson, Johan Herlitz, Örjan Falk, Antonia Panteli, Araz Rawshani

**Affiliations:** a University of Gothenburg, Institute of Medicine, Department of Molecular and Clinical Medicine, Sweden; b Department of Acute Medicine and Geriatrics, Sahlgrenska University Hospital, Gothenburg, Region Västra Götaland, Sweden; c Gothenburg Emergency Medicine Research Group, GEMREG, Sahlgrenska University Hospital, Gothenburg, Sweden; d Department of Cardiology, Sahlgrenska University Hospital, Gothenburg, Region Västra Götaland, Sweden; e The Swedish Cardiopulmonary Resuscitation Registry, Centre of Registries, Västra Götaland, Gothenburg, Sweden; f University of Gothenburg, Institute of Psychiatry, Sweden; g Department of Psychiatry, Sahlgrenska University Hospital, Gothenburg, Region Västra Götaland, Sweden

**Keywords:** Out-of-hospital cardiac arrest (OHCA), Young adults, 30-day survival, Emergency medicine, Heart disease, Psychiatric disease, Psychiatry, Cardiovascular disease, Cardiology

## Abstract

**Background/purpose:**

Individuals with psychiatric conditions have increased cardiovascular morbidity and mortality, yet outcomes following out-of-hospital cardiac arrest (OHCA) in this population remain insufficiently characterized. We aimed to investigate the association between pre-existing psychiatric conditions and outcomes after OHCA in a nationwide Swedish cohort.

**Materials and methods:**

We conducted a registry-based cohort study including all individuals ≥16 years with OHCA reported to the Swedish Registry for Cardiopulmonary Resuscitation (2010–2020). Psychiatric conditions were identified using ICD-10 codes and categorized into major diagnostic groups. The primary outcome was 30-day survival. Secondary outcomes included neurological status at discharge (CPC score) and long-term survival. Logistic regression models adjusted for age and sex were used, and analyses were stratified by initial rhythm.

**Results:**

Among 53,981 individuals, 13,777 (26%) had a prior psychiatric diagnosis. Cardiac arrests in individuals with psychiatric conditions were less often witnessed and less likely to present with a shockable rhythm, despite similar rates of bystander CPR. Intoxication and suicide were markedly more common presumed causes, particularly among younger adults, in whom over half had a psychiatric diagnosis. Thirty-day survival was lower in individuals with psychiatric conditions (9% vs. 12%; adjusted OR 0.52, 95% CI 0.48–0.56). In stratified analyses, this association was evident in shockable but not non-shockable rhythms. Neurological outcomes among survivors were largely comparable.

**Conclusion:**

Pre-existing psychiatric conditions are associated with lower survival after OHCA, largely driven by differences in arrest characteristics and etiology rather than bystander response. These findings highlight a vulnerable population and underscore the importance of targeted prevention and early recognition strategies. Keywords: Out-of-Hospital Cardiac Arrest (OHCA), Psychiatric diagnosis, Young Adults, 30-day survival, Swedish Cardiopulmonary Registry (SRCR), Emergency Medicine.

## Highlights

• Individuals with a prior psychiatric diagnosis had substantially lower 30-day and long-term survival following cardiac arrest.
• Psychiatric comorbidity was particularly common among younger patients, in whom more than half had a prior psychiatric diagnosis.
• Lower rates of witnessed arrests and non-shockable rhythms were observed in psychiatric conditions, with similar rates of bystander CPR.

## Introduction

Individuals with psychiatric conditions have a markedly shorter life expectancy compared to those without psychiatric conditions.^1^, ^2^, ^3^ While the extent of excess mortality varies across studies and diagnoses,^2^, ^4^, ^5^ life expectancy has been shown to be up to 20 years shorter in patients with psychiatric conditions compared to the general population.^2^, ^6^ People with psychiatric conditions have an increased risk of cardiovascular disease (CVD) and CVD related death compared to the general population.^7^, ^8^ The excess mortality is partly explained by CVD, pulmonary disease, infections and neoplasms, but also by external factors such as suicide, accidents, and homicide.^5^, ^6^, ^9^, ^10^

There is limited literature describing the characteristics and outcomes in out-of-hospital cardiac arrest (OHCA) among individuals with pre-existing psychiatric conditions.^11^ Several studies show that patients with psychiatric conditions are more likely to have unwitnessed cardiac arrests and thus less likely to receive bystander cardiopulmonary resuscitation (CPR), as well as presenting with a shockable initial rhythm.^12^, ^13^

We studied characteristics and outcomes after OHCAs in Sweden 2010–2020, to investigate whether previously diagnosed psychiatric conditions were associated with worse outcomes. We additionally focused on younger adults (16–49 years of age) with the aim to examine whether there were differences in characteristics and outcomes in the younger group compared to the entire adult population of OHCA.

## Materials and methods

### Ethical approval

Ethical approval was obtained by the Swedish Ethical Review Authority (2019–01094).

### Registries

The data for this study were obtained from several Swedish registries. The Swedish Registry for Cardiopulmonary Resuscitation (SRCR) is a national quality registry, documenting cases of OHCA since 1990, and in-hospital cardiac arrest (IHCA) since 2006. The emergency medical services (EMS) regularly report treated OHCA, while follow up is mainly conducted by trained nurses and physicians within the hospital system, following the Utstein reporting guidelines.^14^ Over time, data completeness has improved, achieving an ascertainment level exceeding 90% since 2010. The registry and its variables have been previously described.^15^, ^16^

The National Board of Health and Welfare in Sweden manages several nationwide, individual-level databases. These include the Swedish Inpatient Registry and Outpatient Registry, which capture all primary and secondary discharge diagnoses in both inpatient and specialized outpatient care nationwide. The Inpatient Registry includes hospitalizations since 1987 while the Outpatient Registry contains outpatient visits since 2002.^17^ Both registries employed the 10th revision of the International Classification of Disease (ICD) in 1997.

The LISA (longitudinal integrated database for health insurance and labor market studies) registry provides detailed data on health insurance, parental insurance, and unemployment insurance at the individual level. Data is available from 1990 for individuals aged 16 years or older. The Cause of death Registry, established in 1997, uses the 10th revision of the ICD to compile comprehensive statistics on the causes of death.

### Study population

The study population included all individuals aged 16 years or older, corresponding to the minimum age for adult specialized health care in Sweden, who experienced an OHCA reported to the SRCR between 2010 and 2020 (Supplementary Fig. 1). All data was anonymized before analysis. Individuals were stratified according to pre-existing (i.e., a diagnosis established prior to cardiac arrest) psychiatric condition or no pre-existing psychiatric condition, according to ICD10 classification of mental and behavioral disorders.^18^

Psychiatric conditions were classified as any of the following: (1) psychotic disorders (ICD F20–F29); (2) mood disorders (ICD F30–F39), (3) psychoactive substance use disorders (ICD F10–F19), or (4) other psychiatric disorders (F40–F69, F90–F98) (Supplementary Fig. 1). Although individuals could receive multiple ICD diagnoses, each patient was assigned to a single category based on a hierarchy of severity (descending from category 1 to 4). Intellectual disabilities and other developmental disabilities as well as dementia and non-specific mental disorder were not classified as psychiatric conditions. Diagnoses were retrieved from the Inpatient- and Outpatient registries, and data was linked to the registries to provide information on relevant baseline characteristics. The ten most prevalent co-existing conditions were presented, in addition to the circumstances surrounding the cardiac arrest reported in the SRCR (Table 1).

**Table 1:** Baseline characteristics and cardiac arrest-related factors in 53,981 OHCA patients, stratified by age and psychiatric comorbidity.

	**All patients**		**Young patients (16–49)**	
**Characteristic**	**No psychiatric diagnosis**	**Prior psychiatric diagnosis**	**No psychiatric diagnosis**	**Prior psychiatric diagnosis**
	***n* = 40,204**^a^	***n* = 13,777**^a^	***n* = 2768**^a^	***n* = 3284**^a^
Age	75 (65, 83)	65 (50, 75)	40 (29, 46)	35 (27, 43)
Women	13,166 (33%)	5204 (38%)	748 (27%)	1023 (31%)
Born abroad	5685 (14%)	2056 (15%)	574 (21%)	430 (13%)
**Coexisting conditions**				
Essential hypertension	18,741 (47%)	5716 (41%)	227 (8.2%)	271 (8.3%)
Heart failure	9583 (24%)	2823 (20%)	96 (3.5%)	103 (3.1%)
Chronic ischemic heart disease	8810 (22%)	2435 (18%)	64 (2.3%)	64 (1.9%)
Atrial fibrillation or flutter	8940 (22%)	2257 (16%)	61 (2.2%)	59 (1.8%)
Type 2 diabetes mellitus	7847 (20%)	2576 (19%)	105 (3.8%)	159 (4.8%)
Polyosteoarthritis	6645 (17%)	1975 (14%)	65 (2.3%)	103 (3.1%)
Disorders of lipoprotein metabolism and other lipidemias	6556 (16%)	2004 (15%)	78 (2.8%)	85 (2.6%)
Angina pectoris	6572 (16%)	1771 (13%)	43 (1.6%)	36 (1.1%)
Epilepsy and recurrent seizures	5068 (13%)	3032 (22%)	300 (11%)	726 (22%)
Neoplasm of uncertain behavior of oral cavity and digestive organs	5950 (15%)	1873 (14%)	112 (4.0%)	131 (4.0%)
**Presumed cause of cardiac arrest**				
Heart disease	24,772 (69%)	5527 (46%)	896 (37%)	362 (12%)
Overdose or intoxication	168 (0.5%)	1217 (10%)	109 (4.4%)	973 (33%)
Trauma or accident	806 (2.2%)	286 (2.4%)	260 (11%)	112 (3.8%)
Pulmonary disease	1917 (5.3%)	794 (6.6%)	75 (3.1%)	53 (1.8%)
Suffocation	656 (1.8%)	572 (4.7%)	61 (2.5%)	120 (4.1%)
Suicide	325 (0.9%)	757 (6.3%)	168 (6.8%)	487 (17%)
Drowning	229 (0.6%)	140 (1.2%)	72 (2.9%)	50 (1.7%)
Other	6972 (19%)	2819 (23%)	813 (33%)	765 (26%)
Unknown	4359	1665	314	362
**Location of cardiac arrest**				
Home	28,480 (71%)	10,011 (73%)	1523 (55%)	2198 (67%)
Public place	6772 (17%)	1928 (14%)	861 (31%)	595 (18%)
Other places	4791 (12%)	1771 (13%)	372 (13%)	475 (15%)
Unknown	161	67	12	16
**Prehospital interventions**				
Bystander CPR	21,073 (54%)	7392 (56%)	1687 (63%)	1911 (61%)
Unknown	1448	549	89	126
Defibrillated	14,408 (37%)	3016 (23%)	1072 (40%)	551 (18%)
Unknown	1435	655	92	161
Adrenaline	31,441 (79%)	10,723 (79%)	2046 (75%)	2503 (77%)
Unknown	475	179	38	41
**Presentation on EMS arrival**				
**Initial rhythm**				
VF/pVT	9447 (27%)	1675 (14%)	784 (32%)	269 (9.7%)
PEA	6343 (18%)	1900 (16%)	276 (11%)	278 (10.0%)
Asystole	19,844 (56%)	8504 (70%)	1378 (57%)	2240 (80%)
Unknown	4570	1698	330	497
Consciousness on EMS arrival at scene	4450 (11%)	1128 (8.4%)	239 (8.8%)	169 (5.3%)
Unknown	1020	402	64	92
ROSC at arrival at hospital	10,492 (45%)	3266 (44%)	826 (41%)	790 (40%)
Unknown	17,032	6401	775	1324
Witnessed cardiac arrest	26,789 (68%)	7303 (55%)	1612 (60%)	1150 (37%)
Unknown	1082	487	101	145
**Critical time intervals**				
Time from arrest to CPR start	3 (0, 10)	3 (0, 10)	2 (0, 8)	3 (0, 10)
Unknown	8149	3566	639	1008
Time from arrest to defibrillation	15 (8, 23)	16 (9, 26)	13 (7, 20)	18 (10, 31)
Unknown	27,803	11,252	1853	2830
Time from arrest to ROSC	15 (9, 23)	15 (9, 23)	14 (9, 22)	15 (10, 23)
Unknown	29,547	10,184	2023	2509
Time from alarm to EMS arrival	10 (7, 16)	10 (7, 16)	10 (7, 16)	10 (6, 15)
Unknown	5162	1892	389	450

Data presented as median [Q1–Q3] or *n* (%).

a Median (Q1, Q3); *n* (%).

### Exposures

The main exposure was defined as the presence of any pre-existing psychiatric condition, and the secondary exposure was the disease-specific subgroup, classified as described above.

### Subgroup analyses

A subgroup of younger adults (aged 16–49) was analyzed separately and stratified according to any previously diagnosed psychiatric condition (Table 1). From a clinical perspective, individuals under the age of 50 who experience OHCA are considered young; therefore, 49 years was chosen as an upper age limit.

We analyzed characteristics and outcomes according to disease-specific subgroups. Long-term survival in individuals with and without pre-existing psychiatric conditions was analyzed in the subgroup who survived 30 days.

### Outcomes

The primary outcome measure was 30-day survival. Secondary outcome measures were neurological outcome at discharge according to cerebral performance category (CPC-score),^19^ and long-term survival. CPC-score 1–2 was defined as a good neurological outcome.

### Statistical analyses

**Fig. 1:**
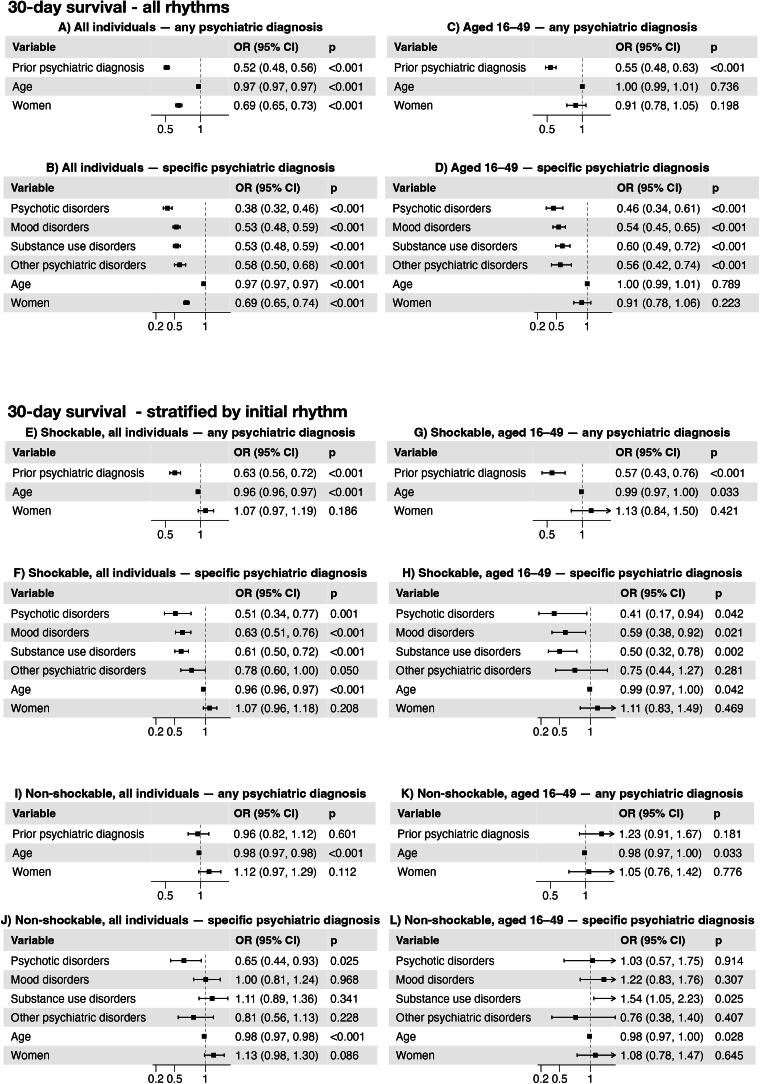
**This figure presents adjusted odds ratios (ORs) with 95% confidence intervals (CIs) from logistic regression models evaluating the association between prior psychiatric disorders and 30-day survival after out-of-hospital cardiac arrest (OHCA), adjusted for age and sex. Patients without prior psychiatric diagnoses served as reference category**.

**Fig. 2:**
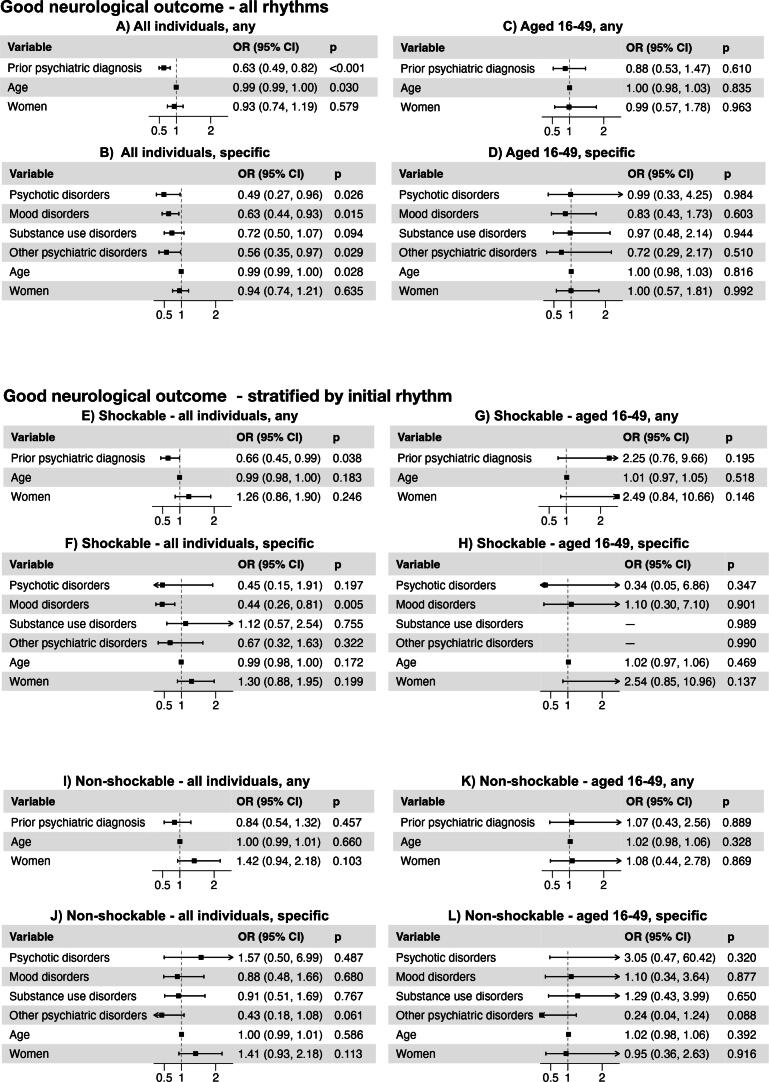
**Adjusted odds ratios (ORs) with 95% confidence intervals (CIs) from logistic regression models evaluating the association between prior psychiatric disorders and good neurological outcome defined as cerebral performance category (CPC-score) 1–2. Patients without prior psychiatric diagnoses served as reference category. In panel (H), categories with missing odds ratios had few events, which yielded very wide confidence intervals, outside the range of the plot**.

30-day survival (Fig. 1) and CPC score (Fig. 2) were analyzed using logistic regression. The presence of any pre-existing psychiatric diagnosis was the primary exposure variable in all models. A directed acyclic graph (Supplementary Fig. 2) was used to identify variables considered relevant for confounding control. Based on this framework, age and sex were included in the adjusted model. Age was modeled as a continuous variable. We tested an interaction term between sex and psychiatric condition in order to explore whether the association between psychiatric disease and outcomes were modified by sex; the interaction term was non-significant. Coexisting conditions and variables related to the resuscitation process (witnessed status, bystander CPR, initial rhythm, location, and EMS response time) were considered to lie on the pathway between exposure and outcome and were therefore not included in the primary analysis. As this was not a hypothesis-driven study aimed at estimating causal effects, but rather to assess associations, the selection of covariates should be interpreted as guided by theoretical considerations and experience rather than strict causal inference.

**Fig. 3:**
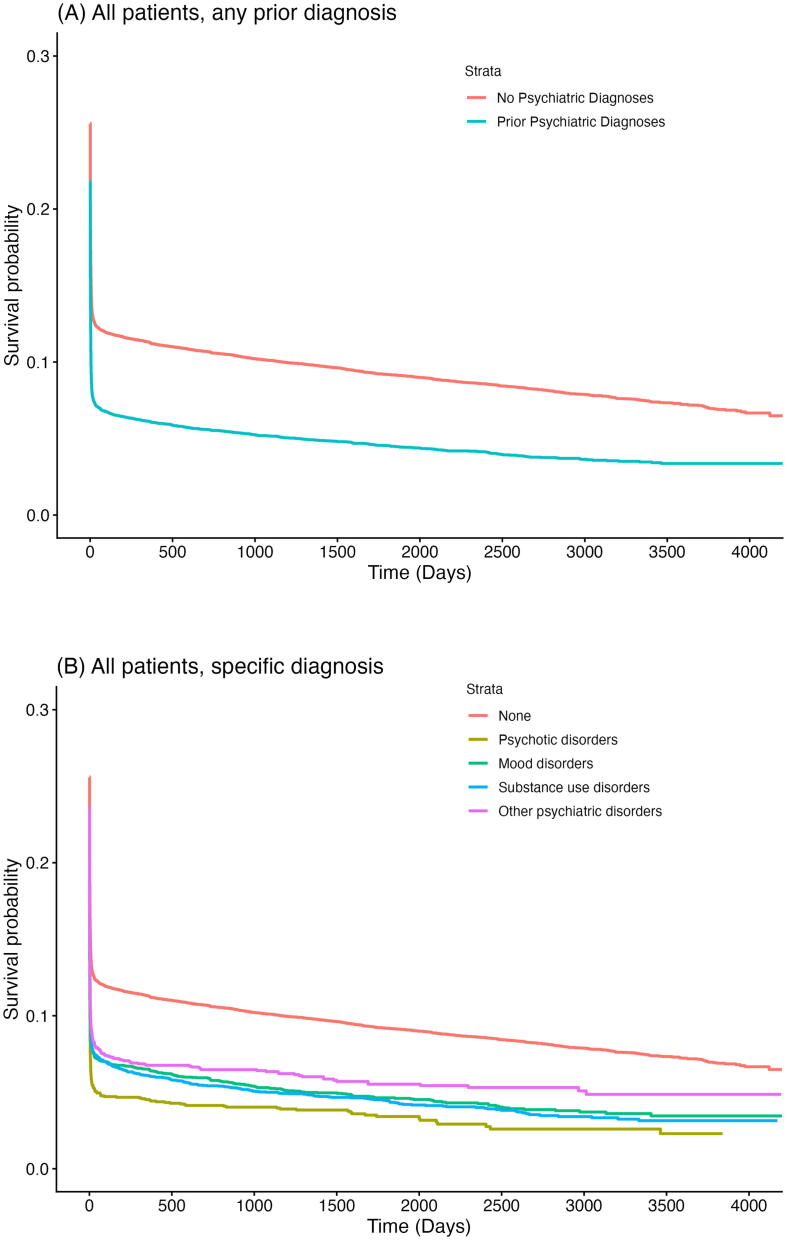

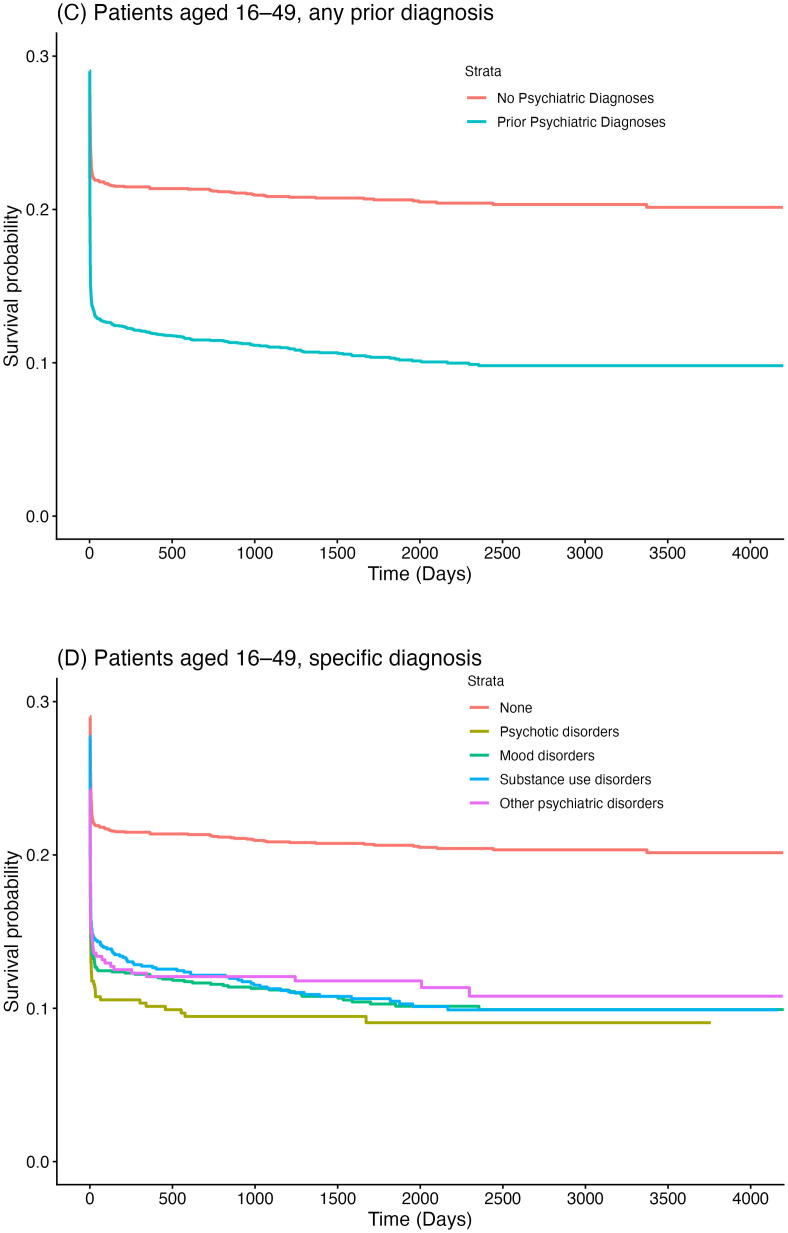
**(A) Kaplan-Meier survival curve after OHCA in adults (≥16 years), stratified by history of prior psychiatric diagnosis. (B) Kaplan-Meier survival curve after OHCA in adults (≥16 years), stratified by history of specific psychiatric diagnosis. (C) Kaplan-Meier survival curve after OHCA in young adults (16–49 years), stratified by history of prior psychiatric diagnosis. (D) Kaplan-Meier survival curve after OHCA in adults (16–49 years), stratified by history of specific psychiatric diagnosis**.

In addition to the primary analyses, we performed stratified analyses according to initial rhythm (shockable vs. non-shockable) given its strong association with survival. Long-term survival was evaluated using Kaplan–Meier estimators (Fig. 3).

All analyses were conducted in R®, version 4.3.2.

### Handling of missing data

Missing data were not imputed, given our assumption that they were missing completely at random and to avoid the potential introduction of bias. The extent of missingness is detailed in Table 1.

## Results

The clinical characteristics and cardiac arrest-related factors among 53,981 individuals registered in the SRCR 2010–2020, including 6052 young adults (16–49 years old) are presented in Table 1. Supplementary Table 1 shows baseline characteristics, presumed cause of cardiac arrest, and variables associated with the cardiac arrest, in relation to specific psychiatric diagnoses.

### Characteristics and cardiac arrest-related factors – all individuals

13,777 out of 53,981 individuals had a psychiatric diagnosis prior to the cardiac arrest. These individuals were younger than those without such a diagnosis (mean age 65 vs. 75 years), and the proportion of women was higher (38% vs. 33%). The distribution of most comorbidities was similar across groups, with the exception of epilepsy, which was more prevalent among individuals with a psychiatric diagnosis, while a slightly higher prevalence of cardiac-related conditions was observed among those without a prior psychiatric diagnosis.

Heart disease was the leading presumed cause of cardiac arrest in both groups, though it was more prevalent in individuals without a pre-existing psychiatric diagnosis. Overdose or intoxication, suffocation, and attempted suicide were more common in the group with a psychiatric diagnosis. The majority of cardiac arrests occurred at home; however, public locations were more frequent among individuals without a prior psychiatric diagnosis. There were no differences between groups in the rate of bystander CPR or administration of adrenaline. Asystole was the initial rhythm in 70% of individuals with a pre-existing psychiatric condition, compared with 56% in those without. In contrast, ventricular fibrillation/pulseless ventricular tachycardia (VF/pVT) was observed twice as frequently in individuals without a psychiatric diagnosis (27%), who were also more likely to receive defibrillation.

### Characteristics and cardiac arrest-related factors – young adults

Among the younger adults, it was more common to have a previous psychiatric diagnosis than not (3284 compared to 2768), and the median age was lower among the individuals with a prior psychiatric diagnosis (35 years compared to 40 years). Coexisting conditions were less common in the younger group but showed the same pattern, with only epilepsy being more common in individuals with a prior psychiatric diagnosis.

Among young individuals without a psychiatric diagnosis, heart disease was the most common presumed cause of cardiac arrest. In contrast, overdose or intoxication represented the leading cause among those with a psychiatric diagnosis, followed by suicide. In this group, 33% of arrests were attributed to intoxication and 17% to suicide, compared with 4.4% and 6.8%, respectively, among those without a psychiatric diagnosis. Trauma and accidents were also more frequent in the younger age group, particularly among individuals without a psychiatric diagnosis.

Among younger individuals, cardiac arrests in public locations were more common in those without a prior psychiatric diagnosis, although rates of bystander CPR did not differ between groups. Individuals without a psychiatric diagnosis were more likely to present with VF/pVT, receive defibrillation, and have a shorter time to first defibrillation compared with those with a prior psychiatric diagnosis.

### 30-day survival

Crude data on 30-day survival showed that 9% (*n* = 1229) of all individuals with a pre-existing psychiatric condition were alive at 30 days and 8% (*n* = 1052) at 1 year, compared to 12% (*n* = 4635) and 10% (*n* = 4188), respectively, of the individuals without a prior psychiatric diagnosis (Supplementary Table 2). In the younger age category, 14% (*n* = 447) of those with a previous psychiatric diagnosis were alive at 30 days, and 12% at 1 year, compared to 22% (*n* = 620) and 22% in those without. In relation to specific psychiatric diagnoses (Supplementary Table 3), psychotic disorders were associated with the poorest survival outcomes.

Fig. 1 shows adjusted analyses of 30-day survival, in all individuals (Fig. 1A, B), and for young adults (Fig. 1C, D). Prior psychiatric diagnosis was associated with reduced odds of 30-day survival after OHCA in all analyses compared to individuals with no previous psychiatric diagnosis. In the overall population, OR was 0.52 (95% CI 0.48, 0.56) with similar estimates across diagnostic subgroups. In the younger age group (Fig. 1C, D), this association remained robust and statistically significant, both for any psychiatric disorder (OR 0.55) and across all diagnostic subgroups. Age and sex were not significant predictors in this subgroup.

Stratified analyses (Fig. 1E–L) show that a prior psychiatric diagnosis was associated with worse survival among individuals with shockable rhythms (Fig. 1E–H). In individuals with non-shockable rhythms (I-L), there was no significant association between prior psychiatric diagnosis and survival.

### CPC-score

Fig. 2 shows CPC-score, adjusted for age and sex, among survivors in relation to prior psychiatric diagnoses. With the exception of substance use disorder, individuals with a prior psychiatric diagnosis had a lower chance of a favorable neurological outcome (CPC 1–2) throughout, as compared to individuals without prior psychiatric diagnoses. There was no difference in neurologic outcome in the younger age category (Fig. 1C, D).

In the stratified analyses the association between prior psychiatric diagnosis and worse neurological outcome was weaker, yet significant in shockable rhythms. In all other analyses, the association was non-significant.

### Long-term survival

Fig. 3 shows Kaplan-Meier survival curves for all patients stratified by psychiatric history. Patients with a prior psychiatric diagnosis displayed consistently lower survival probabilities compared to those without such a diagnosis, across all age groups (Fig. 3A–D).

## Discussion

In this nationwide study of out-of-hospital cardiac arrest (OHCA), individuals with a prior psychiatric diagnosis had substantially lower 30-day survival compared with those without such conditions. This pattern was consistent across age groups, including younger adults, among whom more than half had a prior psychiatric diagnosis. With the exception of epilepsy, comorbidity patterns were otherwise similar between groups, suggesting broadly comparable cardiovascular risk profiles.

These findings may, in part, be explained by differences in arrest characteristics. Although rates of bystander CPR were similar between groups, individuals with psychiatric conditions were less likely to have a witnessed arrest and less likely to present with a shockable initial rhythm – two of the strongest predictors of survival following OHCA – which is consistent with previous research.^13^, ^15^, ^20^ Together, these findings point towards important differences in the circumstances surrounding the OHCA that may contribute to the observed survival disparities.

The presumed etiology of the cardiac arrest also differed substantially. While heart disease remained the most common presumed cause overall, intoxication and suicide were markedly more frequent among individuals with psychiatric conditions, particularly in younger adults. In this subgroup, intoxication alone accounted for one-third of cases, with suicide representing an additional substantial proportion. These etiologies are often associated with hypoxic mechanisms and a lower likelihood of shockable rhythms, which may partly explain the poorer outcomes observed. Other factors, such as differences in health behaviors, access to care, or prior medical management, may also contribute to the lower survival observed, although these were not directly assessed in our study.

The association between mental health issues and substance abuse is well established,^21^, ^22^ whereas outcomes after OHCA in this population remain less well studied. A Danish nationwide study reported substantially lower 30-day survival among individuals with psychiatric conditions, even after accounting for comorbidity burden and arrest characteristics.^23^ A recent nationwide Scottish cohort study similarly demonstrated lower survival among individuals with pre-existing psychiatric conditions following OHCA, along with less favorable arrest characteristics, including a lower prevalence of shockable rhythms.^24^ Importantly, the Danish study also identified differences in pre-hospital care, including lower rates of bystander CPR and witnessed arrests. In contrast, while we similarly observed fewer witnessed arrests and a lower prevalence of shockable rhythms among individuals with psychiatric conditions, we did not find differences in bystander CPR rates. This discrepancy may reflect temporal improvements in public CPR awareness, differences in population characteristics, or variations in emergency response systems between settings. Despite comparable rates of bystander CPR, individuals with psychiatric conditions in our cohort were less likely to receive defibrillation, suggesting that arrest characteristics and underlying etiology may play a more prominent role than differences in bystander response in explaining survival disparities.

In stratified analyses according to initial rhythm, the association between psychiatric conditions and survival was evident among individuals with shockable rhythms but not among those with non-shockable rhythms. This suggests that the excess mortality associated with psychiatric conditions may be most pronounced in potentially treatable cardiac arrests, whereas outcomes in non-shockable rhythms are uniformly poor regardless of psychiatric status. In the younger cohort, the association between psychiatric conditions and survival was attenuated within strata of initial rhythm, further supporting the notion that differences in arrest characteristics – particularly the lower prevalence of shockable rhythms – are key contributors to the observed disparities.

Neurological outcomes among survivors were largely similar between groups. Although individuals with psychiatric conditions had a lower likelihood of favorable neurological outcome overall, this difference was attenuated in stratified analyses and was not observed in younger adults. These findings suggest that among those who survive the initial event, the potential for neurological recovery may be broadly comparable regardless of psychiatric status.

Overall, individuals with psychiatric conditions had consistently lower survival following OHCA, with differences emerging early and persisting over time. These findings identify a vulnerable population with poorer outcomes and highlight the importance of further research to better understand modifiable factors across the chain of survival, particularly those related to arrest recognition, underlying etiology, and early resuscitation.

### Limitations

Several limitations should be acknowledged. First, uncertainties remain regarding the circumstances surrounding the OHCA, including the presumed etiology and critical time intervals, which may be subject to misclassification or measurement error. As in most registry-based studies, we consider such misclassification to be largely non-differential; however, it may still have introduced uncertainty into the estimates.

Second, residual confounding cannot be excluded. We did not account for several potentially important factors, including socioeconomic status, medication use (e.g., psychotropic drugs), and pre-arrest functional status, all of which may influence both the likelihood of psychiatric diagnosis and survival outcomes.

Third, psychiatric diagnoses were based on registry data and may be subject to misclassification, particularly for less severe or undiagnosed conditions. In addition, we did not explicitly account for comorbidity between psychiatric illness and substance use disorders, which are closely linked and may have differential effects on outcomes.

Finally, the observational design limits causal inference, and the findings should be interpreted as associations. Although the study was conducted in a nationwide setting with high-quality registry data, the generalizability to other healthcare systems with different emergency response structures or population characteristics may be limited.

## Conclusions

Pre-existing psychiatric conditions are associated with lower survival after OHCA, largely driven by differences in arrest characteristics and etiology rather than bystander response. These findings highlight a vulnerable population and underscore the importance of targeted prevention and early recognition strategies.

## Sources of funding

Swedish Research Council (2019–02019), Swedish state under the agreement between the Swedish government, and the county councils (ALFGBG-971482), The Wallenberg Center for Molecular and Translational Medicine.

## CRediT authorship contribution statement

**Linnea Gustafsson:** Writing – original draft, Visualization, Methodology, Investigation, Formal analysis, Data curation, Conceptualization. **Anna Myredal:** Writing – review & editing, Conceptualization. **Clara Hjalmarsson:** Formal analysis, Data curation. **Lina Holmqvist:** Writing – review & editing, Supervision. **Alfred Hjalmarsson:** Formal analysis, Data curation. **Johan Herlitz:** Writing – review & editing. **Örjan Falk:** Writing – review & editing, Conceptualization. **Antonia Panteli:** Writing – review & editing. **Araz Rawshani:** Writing – review & editing, Supervision, Resources, Formal analysis, Data curation, Conceptualization.

## Declaration of competing interest

None.

## Appendix A Supplementary material

Supplementary Fig. 1 Flowchart of the study population stratified by history of psychiatric diagnosis. *SRCR = The Swedish Registry of Cardiopulmonary Resuscitation. OHCA = Out-of-Hospital Cardiac Arrest*.

Supplementary Fig. 2 Directed acyclic graph (DAG) illustrating the assumed relationships between psychiatric disease, covariates and survival. Age and sex were identified as confounders and included in the adjusted model. Coexisting conditions and variables related to the resuscitation process (witnessed status, bystander CPR, initial rhythm, location and EMS response time) were considered to lie in the causal pathway between exposure and outcome.

Supplementary Table 1 Baseline characteristics and cardiac arrest-related factors in 53,981 OHCA patients, stratified by psychiatric comorbidity.

Supplementary Table 2 Survival after OHCA, stratified by age and psychiatric comorbidity.

Supplementary Table 3 Survival after OHCA, stratified by specific condition.

## References

[1] Walker E.R., McGee R.E., Druss B.G. (2015). Mortality in mental disorders and global disease burden implications: a systematic review and meta-analysis. JAMA Psychiat.

[2] Chesney E., Goodwin G.M., Fazel S. (2014). Risks of all-cause and suicide mortality in mental disorders: a meta-review. World Psychiatry.

[3] Roerecke M., Rehm J. (2013). Alcohol use disorders and mortality: a systematic review and meta-analysis. Addiction.

[4] Correll C.U., Solmi M., Croatto G., Schneider L.K., Rohani-Montez S.C., Fairley L., Smith N., Bitter I., Gorwood P., Taipale H. (2022). Mortality in people with schizophrenia: a systematic review and meta-analysis of relative risk and aggravating or attenuating factors. World Psychiatry.

[5] Oude Voshaar R.C., Aprahamian I., Borges M.K., van den Brink R.H.S., Marijnissen R.M., Hoogendijk E.O., van Munster B., Jeuring H.W. (2021). Excess mortality in depressive and anxiety disorders: the lifelines cohort study. Eur Psychiatry.

[6] Nordentoft M., Wahlbeck K., Hällgren J., Westman J., Osby U., Alinaghizadeh H., Gissler M., Laursen T.M. (2013). Excess mortality, causes of death and life expectancy in 270,770 patients with recent onset of mental disorders in Denmark, Finland and Sweden. Plos One.

[7] Correll C.U., Solmi M., Veronese N., Bortolato B., Rosson S., Santonastaso P., Thapa-Chhetri N., Fornaro M., Gallicchio D., Collantoni E. (2017). Prevalence, incidence and mortality from cardiovascular disease in patients with pooled and specific severe mental illness: a large-scale meta-analysis of 3,211,768 patients and 113,383,368 controls. World Psychiatry.

[8] Lambert A.M., Parretti H.M., Pearce E., Price M.J., Riley M., Ryan R., Tyldesley-Marshall N., Avşar T.S., Matthewman G., Lee A. (2022). Temporal trends in associations between severe mental illness and risk of cardiovascular disease: a systematic review and meta-analysis. PLoS Med.

[9] de Mooij L.D., Kikkert M., Theunissen J., Beekman A.T.F., de Haan L., Duurkoop P., Van H.L., Dekker J.J.M. (2019). Dying too soon: excess mortality in severe mental illness. Front Psychiatry.

[10] Hiroeh U., Appleby L., Mortensen P.B., Dunn G. (2001). Death by homicide, suicide, and other unnatural causes in people with mental illness: a population-based study. Lancet.

[11] Alotaibi R., Halbesma N., Bijman L.A.E., Clegg G., Smith D.J., Jackson C.A. (2022). Incidence, characteristics and outcomes of out-of-hospital cardiac arrests in patients with psychiatric illness: a systematic review. Resusc Plus.

[12] Ko D.T., Qiu F., Koh M., Dorian P., Cheskes S., Austin P.C., Scales D.C., Wijeysundera H.C., Verbeek P.R., Drennan I. (2016). Factors associated with out-of-hospital cardiac arrest with pulseless electric activity: a population-based study. Am Heart J.

[13] Ishida T., Sugiyama K., Tanabe T., Hamabe Y., Mimura M., Suzuki T., Uchida H. (2020). Lower proportion of fatal arrhythmia in sudden cardiac arrest among patients with severe mental illness than nonpsychiatric patients. Psychosomatics.

[14] Perkins G.D., Jacobs I.G., Nadkarni V.M., Berg R.A., Bhanji F., Biarent D., Bossaert L.L., Brett S.J., Chamberlain D., de Caen A.R. (2015). Cardiac arrest and cardiopulmonary resuscitation outcome reports: update of the Utstein Resuscitation Registry Templates for Out-of-Hospital Cardiac Arrest: a statement for healthcare professionals from a task force of the International Liaison Committee on Resuscitation (American Heart Association, European Resuscitation Council, Australian and New Zealand Council on Resuscitation, Heart and Stroke Foundation of Canada, InterAmerican Heart Foundation, Resuscitation Council of Southern Africa, Resuscitation Council of Asia); and the American Heart Association Emergency Cardiovascular Care Committee and the Council on Cardiopulmonary, Critical Care, Perioperative and Resuscitation. Circulation.

[15] Hasselqvist-Ax I., Riva G., Herlitz J., Rosenqvist M., Hollenberg J., Nordberg P., Ringh M., Jonsson M., Axelsson C., Lindqvist J. (2015). Early cardiopulmonary resuscitation in out-of-hospital cardiac arrest. N Engl J Med.

[16] Strömsöe A., Svensson L., Axelsson Å.B., Göransson K., Todorova L., Herlitz J. (2013). Validity of reported data in the Swedish Cardiac Arrest Register in selected parts in Sweden. Resuscitation.

[17] Ludvigsson J.F., Andersson E., Ekbom A., Feychting M., Kim J.L., Reuterwall C., Heurgren M., Olausson P.O. (2011). External review and validation of the Swedish national inpatient register. BMC Public Health.

[18] World Health Organization. International statistical classification of diseases and related health problems, 10th Revision; 2019. https://icd.who.int/browse10/2019/en [Accessed January 5].

[19] Edgren E., Hedstrand U., Kelsey S., Sutton-Tyrrell K., Safar P. (1994). Assessment of neurological prognosis in comatose survivors of cardiac arrest. BRCT I Study Group. Lancet.

[20] Sasson C., Rogers M.A., Dahl J., Kellermann A.L. (2010). Predictors of survival from out-of-hospital cardiac arrest: a systematic review and meta-analysis. Circ Cardiovasc Qual Outcomes.

[21] Jones C.M., McCance-Katz E.F. (2019). Co-occurring substance use and mental disorders among adults with opioid use disorder. Drug Alcohol Depend.

[22] Schuckit M.A. (2006). Comorbidity between substance use disorders and psychiatric conditions. Addiction.

[23] Barcella C.A., Mohr G.H., Kragholm K., Blanche P., Gerds T.A., Wissenberg M., Hansen S.M., Bundgaard K., Lippert F.K., Folke F. (2019). Out-of-hospital cardiac arrest in patients with psychiatric disorders – characteristics and outcomes. Resuscitation.

[24] Alotaibi R. (2026). A nationwide Scottish cohort study of the association between pre-existing mental illness and out-of-hospital cardiac arrest survival. Resusc Plus.

