# Supplementary material for: Characteristics and outcomes after out-of-hospital cardiac arrests in individuals with pre-existing psychiatric conditions, compared to those without
Source: Resusc Plus. 2026 May 6;29:101356. doi: 10.1016/j.resplu.2026.101356 (PMC13214532; doi:10.1016/j.resplu.2026.101356)
Supplement: Supplementary Fig. 2 — Directed acyclic graph (DAG) illustrating the assumed relationships between psychiatric disease, covariates and survival. Age and sex were identified as confounders and included in the adjusted model. Coexisting conditions and variables related to the resuscitation process (witnessed status, bystander CPR, initial rhythm, location and EMS response time) were considered to lie in the causal pathway between exposure and outcome. [file mmc2.docx]

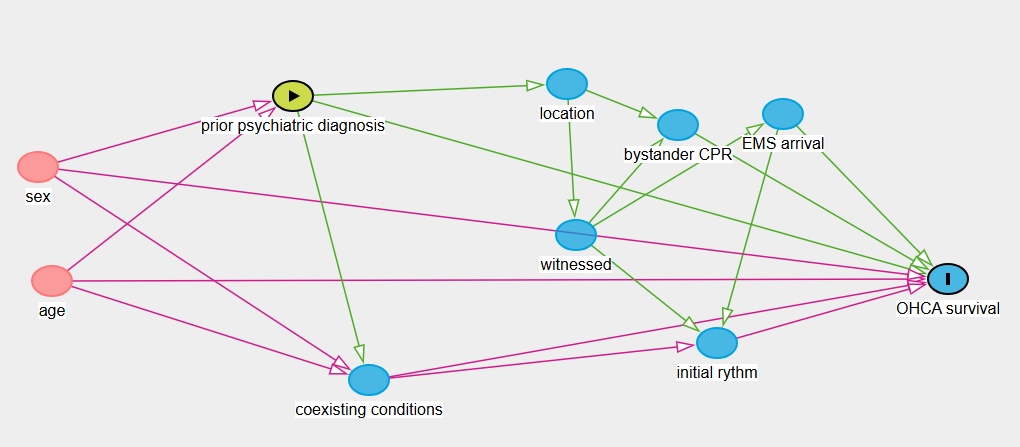


Supplementary Figure 2. Directed acyclic graph (DAG) illustrating the assumed relationships between psychiatric disease, covariates and survival. Age and sex were identified as confounders and included in the adjusted model. Coexisting conditions and variables related to the resuscitation process (witnessed status, bystander CPR, initial rhythm, location and EMS response time) were considered to lie in the causal pathway between exposure and outcome.
