# Supplementary material for: Characteristics and outcomes after out-of-hospital cardiac arrests in individuals with pre-existing psychiatric conditions, compared to those without
Source: Resusc Plus. 2026 May 6;29:101356. doi: 10.1016/j.resplu.2026.101356 (PMC13214532; doi:10.1016/j.resplu.2026.101356)
Supplement: Supplementary Table 1 — Baseline characteristics and cardiac arrest-related factors in 53,981 OHCA patients, stratified by psychiatric comorbidity. [file mmc3.docx]

Supplementary Table 1. Baseline Characteristics and cardiac arrest-related factors in 53,981 OHCA patients, stratified by psychiatric comorbidity.

Data presented as median [Q1–Q3] or n (%)

|  | **None** | **Psychotic disorders** | **Mood disorders** | **Substance use disorders** | **Other psychiatric disorders** |
| --- | --- | --- | --- | --- | --- |
|  | n = 40,204*^1^* | n = 1,822*^1^* | n = 4,996*^1^* | n = 4,884*^1^* | n = 2,075*^1^* |
| Age | 75 (65, 83) | 61 (49, 71) | 65 (49, 77) | 65 (53, 73) | 68 (52, 79) |
| Women | 13,166 (33%) | 780 (43%) | 2,257 (45%) | 1,285 (26%) | 882 (43%) |
| Born abroad | 5,685 (14%) | 359 (20%) | 688 (14%) | 672 (14%) | 337 (16%) |
| **Coexisting Conditions** |  |  |  |  |  |
| Hypertension | 18,741 (47%) | 496 (27%) | 2,187 (44%) | 2,117 (43%) | 916 (44%) |
| Heart Failure | 9,583 (24%) | 242 (13%) | 961 (19%) | 1,102 (23%) | 518 (25%) |
| Chronic Ischemic Heart Disease | 8,810 (22%) | 187 (10%) | 828 (17%) | 997 (20%) | 423 (20%) |
| Atrial Fibrillation or Flutter | 8,940 (22%) | 165 (9.1%) | 775 (16%) | 897 (18%) | 420 (20%) |
| Type 2 Diabetes Mellitus | 7,847 (20%) | 371 (20%) | 931 (19%) | 882 (18%) | 392 (19%) |
| Polyosteoarthritis | 6,645 (17%) | 165 (9.1%) | 835 (17%) | 640 (13%) | 335 (16%) |
| Disorders of lipoprotein metabolism and other lipidemias | 6,556 (16%) | 151 (8.3%) | 695 (14%) | 803 (16%) | 355 (17%) |
| Angina Pectoris | 6,572 (16%) | 116 (6.4%) | 651 (13%) | 676 (14%) | 328 (16%) |
| Epilepsy and recurrent seizures | 5,068 (13%) | 359 (20%) | 1,297 (26%) | 884 (18%) | 492 (24%) |
| Neoplasm of uncertain behavior of oral cavity and digestive organs | 5,950 (15%) | 174 (9.5%) | 685 (14%) | 693 (14%) | 321 (15%) |
| **Presumed Cause of Cardiac Arrest** |  |  |  |  |  |
| Heart disease | 24,772 (69%) | 604 (38%) | 1,945 (44%) | 2,045 (48%) | 933 (51%) |
| Overdose or intoxication | 168 (0.5%) | 164 (10%) | 506 (11%) | 490 (11%) | 57 (3.1%) |
| Trauma or accident | 806 (2.2%) | 35 (2.2%) | 89 (2.0%) | 109 (2.5%) | 53 (2.9%) |
| Pulmonary disease | 1,917 (5.3%) | 94 (5.9%) | 231 (5.2%) | 322 (7.5%) | 147 (8.1%) |
| Suffocation | 656 (1.8%) | 150 (9.4%) | 225 (5.1%) | 122 (2.9%) | 75 (4.1%) |
| Suicide | 325 (0.9%) | 103 (6.5%) | 432 (9.8%) | 129 (3.0%) | 93 (5.1%) |
| Drowning | 229 (0.6%) | 26 (1.6%) | 53 (1.2%) | 37 (0.9%) | 24 (1.3%) |
| Other | 6,972 (19%) | 419 (26%) | 943 (21%) | 1,022 (24%) | 435 (24%) |
| Unknown | 4,359 | 227 | 572 | 608 | 258 |
| **Location of Cardiac Arrest** |  |  |  |  |  |
| Home | 28,480 (71%) | 1,189 (66%) | 3,735 (75%) | 3,557 (73%) | 1,530 (74%) |
| Public place | 6,772 (17%) | 294 (16%) | 590 (12%) | 750 (15%) | 294 (14%) |
| Other places | 4,791 (12%) | 332 (18%) | 645 (13%) | 553 (11%) | 241 (12%) |
| Unknown | 161 | 7 | 26 | 24 | 10 |
| **Prehospital Interventions** |  |  |  |  |  |
| Bystander CPR | 21,073 (54%) | 1,045 (60%) | 2,677 (56%) | 2,513 (54%) | 1,157 (58%) |
| Unknown | 1,448 | 69 | 195 | 193 | 92 |
| Defibrillated | 14,408 (37%) | 273 (16%) | 993 (21%) | 1,175 (25%) | 575 (29%) |
| Unknown | 1,435 | 97 | 233 | 235 | 90 |
| Adrenaline | 31,441 (79%) | 1,424 (79%) | 3,885 (79%) | 3,796 (79%) | 1,618 (79%) |
| Unknown | 475 | 30 | 56 | 67 | 26 |
| **Presentation on EMS Arrival**  Initial Rhythm |  |  |  |  |  |
| VF/pVT | 9,447 (27%) | 128 (8.0%) | 559 (13%) | 676 (16%) | 312 (17%) |
| PEA | 6,343 (18%) | 217 (14%) | 724 (16%) | 634 (15%) | 325 (18%) |
| Asystole | 19,844 (56%) | 1,252 (78%) | 3,117 (71%) | 2,951 (69%) | 1,184 (65%) |
| Unknown | 4,570 | 225 | 596 | 623 | 254 |
| Consciousness on EMS arrival at scene | 4,450 (11%) | 109 (6.2%) | 404 (8.3%) | 413 (8.7%) | 202 (10.0%) |
| Unknown | 1,020 | 61 | 149 | 140 | 52 |
| ROSC on arrival to hospital | 10,492 (45%) | 355 (40%) | 1,180 (45%) | 1,194 (45%) | 537 (46%) |
| Unknown | 17,032 | 928 | 2,346 | 2,218 | 909 |
| Witnessed cardiac arrest | 26,789 (68%) | 892 (51%) | 2,556 (53%) | 2,586 (55%) | 1,269 (63%) |
| Unknown | 1,082 | 63 | 179 | 177 | 68 |
| **Critical Time Intervals** |  |  |  |  |  |
| Time from arrest to CPR start | 3 (0, 10) | 3 (0, 10) | 3 (0, 10) | 4 (0, 10) | 2 (0, 9) |
| Unknown | 8,149 | 452 | 1,319 | 1,309 | 486 |
| Time from arrest to defibrillation | 15 (8, 23) | 17 (10, 30) | 17 (9, 27) | 15 (10, 25) | 15 (9, 27) |
| Unknown | 27,803 | 1,590 | 4,153 | 3,924 | 1,585 |
| Time from arrest to ROSC | 15 (9, 23) | 14 (9, 21) | 15 (9, 23) | 15 (9, 23) | 15 (10, 24) |
| Unknown | 29,547 | 1,376 | 3,703 | 3,606 | 1,499 |
| Time from alarm to EMS arrival | 10 (7, 16) | 9 (6, 15) | 10 (7, 16) | 10 (7, 16) | 10 (7, 16) |
| Unknown | 5,162 | 264 | 659 | 684 | 285 |
| *^1^*Median (Q1, Q3); n (%) | | | | | |
