# Supplementary material for: Characteristics and outcomes after out-of-hospital cardiac arrests in individuals with pre-existing psychiatric conditions, compared to those without
Source: Resusc Plus. 2026 May 6;29:101356. doi: 10.1016/j.resplu.2026.101356 (PMC13214532; doi:10.1016/j.resplu.2026.101356)
Supplement: Supplementary Table 2 — Survival after OHCA, stratified by age and psychiatric comorbidity. [file mmc4.docx]

Supplementary Table 2: Survival after OHCA, stratified by age and psychiatric comorbidity

|  | **All Patients** | | **Young Patients (16-49)** | |
| --- | --- | --- | --- | --- |
|  | **No prior psychiatric diagnosis**  n = 40,204*^1^* | **Prior psychiatric diagnosis**  n =13,777*^1^* | **No prior psychiatric diagnosis**  n = 2,768*^1^* | **Prior psychiatric diagnosis**  n = 3,284*^1^* |
| **Survival at 30 days** |  |  |  |  |
| Alive | 4,635 (12%) | 1,229 (9%) | 620 (22%) | 447 (14%) |
| Dead | 35,569 (88%) | 12,548 (91%) | 2,148 (78%) | 2,837 (86%) |
| **Survival at 365 days** |  |  |  |  |
| Alive | 4,188 (10%) | 1,052 (8%) | 603 (22%) | 405 (12%) |
| Dead | 36,016 (90%) | 12,725 (92%) | 2,165 (78%) | 2,879 (88%) |
| *^1^*n (%) | | | | |
