# Supplementary material for: Characteristics and outcomes after out-of-hospital cardiac arrests in individuals with pre-existing psychiatric conditions, compared to those without
Source: Resusc Plus. 2026 May 6;29:101356. doi: 10.1016/j.resplu.2026.101356 (PMC13214532; doi:10.1016/j.resplu.2026.101356)
Supplement: Supplementary Table 3 — Survival after OHCA, stratified by specific condition. [file mmc5.docx]

Supplementary Table 3: Survival after OHCA, stratified by specific condition

|  | **None** | **Psychotic disorders** | **Mood disorders** | **Substance use disorders** | **Other psychiatric disorders** |
| --- | --- | --- | --- | --- | --- |
|  | N = 40,204*^1^* | n = 1,822*^1^* | n = 4,996*^1^* | n = 4,884*^1^* | n = 2,075*^1^* |
| **Survival at 30 days** |  |  |  |  |  |
| Alive | 4,635 (12%) | 129 (7%) | 446 (9%) | 460 (9%) | 194 (9%) |
| Dead | 35,569 (88%) | 1,693 (32%) | 4,550 (91%) | 4,424 (91%) | 1,881 (91%) |
| **Survival at 365 days** |  |  |  |  |  |
| Alive | 4,188 (10%) | 113 (6%) | 394 (7%) | 382 (8.7%) | 163 (8.0%) |
| Dead | 36,016 (90%) | 1,709 (94%) | 4,602 (93%) | 4,502 (91%) | 1,912 (92%) |
| *^1^*n (%) | | | | | |
